# Swedish Quality Registry for Caries and Periodontal Diseases – a framework for quality development in dentistry

**DOI:** 10.1111/idj.12481

**Published:** 2019-04-18

**Authors:** Inger von Bültzingslöwen, Hans Östholm, Lars Gahnberg, Dan Ericson, Jan L. Wennström, Jörgen Paulander

**Affiliations:** ^1^ Public Dental Service County Council of Värmland Karlstad Sweden; ^2^ Institute of Odontology Sahlgrenska Academy at University of Gothenburg Gothenburg Sweden; ^3^ Division of Oral Diseases Department of Dental Medicine Karolinska Institute Stockholm Sweden; ^4^ Faculty of Odontology Malmö University Malmö Sweden

**Keywords:** Odontology, epidemiology, oral health, big data, quality registry

## Abstract

There is a need for monitoring dental health and healthcare, as support for quality development, allocation of resources and long‐term planning of dental care. The aim of this paper is to describe the concept and implementation of the Swedish Quality Registry for Caries and Periodontal Diseases (SKaPa). **Materials and methods:** The SKaPa receives information by automatic transfer of data daily from electronic patient dental records via secure connections from affiliated dental care organisations (DCOs). The registry stores information about DCOs, dental professionals and patients. Information on a patient level includes personal identifier, gender, age, living area, dental status, risk assessments for caries and periodontitis, and dental care provided. In addition, data generated from a global question on patient‐perceived oral health are uploaded. In total, more than 400 variables are transferred to the registry and updated daily. **Results:** In 2018, all of the 21 public DCOs and the largest private DCO in Sweden were affiliated to SKaPa, representing a total of 1,089 public and 234 private dental clinics. The accumulated amount of information on dental healthcare covers 6.9 million individuals out of the total Swedish population of 10 million. SKaPa produces reports on de‐identified data, both cross‐sectional and longitudinal. **Conclusion:** As a nationwide registry based on automatic retrieval of data directly from patient records, SKaPa offers the basis for a new era of systematic evaluation of oral health and quality of dental care. The registry supports clinical and epidemiological research, data mining and external validation of results from randomised controlled trials

## **Introduction**

National quality registries (NQRs) have been successfully used as providers of pertinent data for quality improvements in general healthcare for over three decades^1^. The NQRs receive and store personalised information on general health, medical interventions and outcomes of treatment in a way that allows data to be compiled and analysed for comparison between groups of patients, different clinics and healthcare providers^2^. The NQRs are considered powerful tools in support of quality development of care and clinical research^3^. In dentistry, however, there has been no NQR available.

To monitor dental health and healthcare through an NQR is warranted for the support of quality development of dental care. Valid data for health economic analyses are also essential for allocation of resources and long‐term planning of dental care^4^. In consideration of these demands, an NQR in dentistry with special reference to the most common oral diseases worldwide^5^ was launched, known as the Swedish Quality Registry for Caries and Periodontal Diseases (SKaPa; *Svenskt kvalitetsregister för Karies och Parodontit* in Swedish). The great majority of Swedish patient record systems in dentistry are electronic and use a personal identifier unique to each Swedish resident^6^. In addition, a national code system is used for documentation of diagnoses/conditions and treatments^7^. Considering that in Sweden approximately 95% of all children and adolescents and about 75% of individuals aged 20 years and older attend a dental clinic on regular basis^8^, data from a large percentage of the population would be available based on automatic retrieval of data from electronic patient records. The aim of this article is to present the concept and implementation of the NQR SKaPa.

## **Objectives of the SKaPa registry**

With the overall goal of supporting the improvement of dental healthcare, the specific objectives of SKaPa are to:

- Produce continuous national, regional and local epidemiological information, both cross‐sectional and longitudinal;
- Support quality development in everyday dentistry;
- Describe dental care related to caries and periodontal diseases;
- Serve as a source for follow‐up of national and regional clinical practice guidelines;
- Serve as a source for dental research and health‐economic evaluation of dental healthcare; and
- Evaluate patient self‐reported oral health and self‐reported treatment outcomes.

## **Organisation**

The SKaPa organisation is structured according to recommendations by the Swedish Association of Local Authorities and Regions (SALAR)^9^. SKaPa is directed by a steering committee, which includes representatives from private and public dental care organisations (DCOs), experts in cariology and periodontology, and a patient representative. A registry manager is in charge of the executive committee and operative staff. Participating DCOs and the main suppliers of dental record systems are represented at an annual user meeting, which serves as a forum for the development of and response to questions regarding the processes and development of the registry.

The SKaPa concept requires powerful computer hardware and software, as well as specialised competencies regarding odontology, science, informatics, statistics and health economics. Considering all costs for running the registry, the calculated annual cost per individual patient data set amounts to less than 0.1 euro.

## **Regulatory issues**

The SKaPa registry is synchronised with the Patient Data Act^10^, and evaluated and supervised by Swedish authorities. Information about the affiliation to the registry must be available to all patients. There is no requirement for a signed consent; however, individual data cannot be collected if the patient opposes it. The patient must also be informed about the right to have his/her data removed from the registry at any time.

## **The concept**

**Figure 1:**
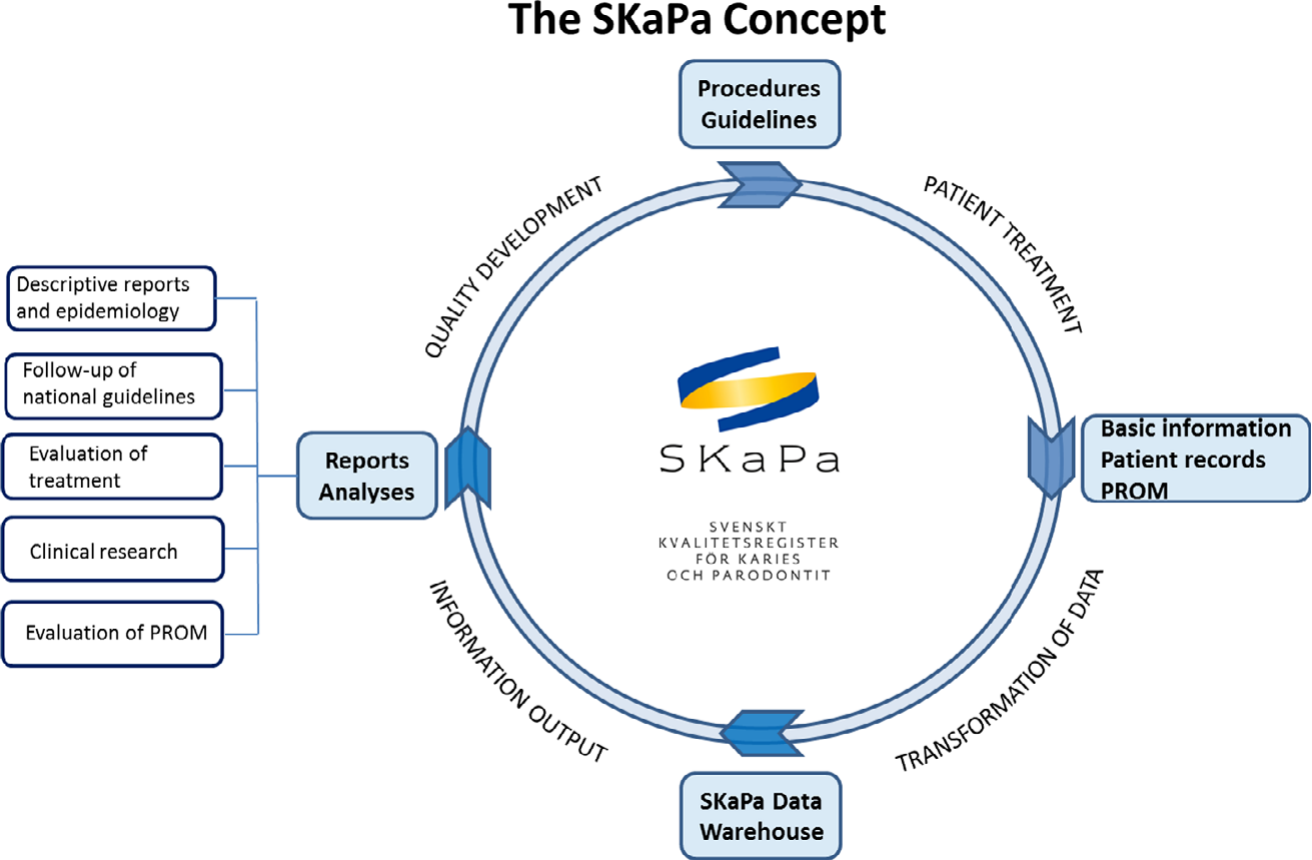
The principal design of the SKaPa concept. PROM, patient‐reported outcome measures.

The principal design of the SKaPa concept is schematically described in *Figure* 1. The registry is built to automatically receive information, according to SKaPa specifications, from the patient data record systems of each DCO. The information is transferred via secure connections to the SKaPa data warehouse (SKaPa DW) on a daily basis. The data warehouse is an SQL (Structured Query Language) relational database, organised in a star structure^11^, allowing efficient addition of new items to the data structure.

The SKaPa DW stores information relating to DCOs, dental professionals and patient dental healthcare. Information at the patient level includes personal identifier, gender, age, living area, risk assessment for caries and periodontal diseases, and dental status based on recordings at tooth/tooth site levels. Risk assessment is automatically calculated by the dental record systems based on an algorithm including a number of variables recorded by the clinician. Dental healthcare provided is reported according to the national coding system for diagnoses/conditions and treatments (TLV; Swedish Dental and Pharmaceutical Benefits Agency)^7^ for primary and permanent dentitions on patient, tooth and tooth surface levels. Furthermore, data on patient‐perceived oral health^12^ are uploaded, using a global question. In total more than 400 variables are transferred to the registry (a complete list of variables can be obtained from the corresponding author).

Data extraction files from the patient records, data loading and evaluation of plausibility of retrieved information are validated and secured before a new DCO is affiliated to SKaPa, as well as on a continuous and regular basis, comparing original patient records with output data from SKaPa.

## **Affiliated dental care organisations and population coverage**

Since the launch of the registry in 2008, all 21 public DCOs in Sweden, representing 1,089 public dental clinics, have been affiliated to SKaPa, as well as the largest private DCO, which currently has 234 of their dental clinics connected. Care performed by approximately 8,800 dentists and 4,000 dental hygienists is so far included. Data on dental care for all patients attending the affiliated DCOs will automatically be accessible for delivery to SKaPa. The accumulated number of individuals included in the SKaPa DW has increased from approximately 261,000 in 2009 to 6.9 million in 2018 (*Table* 1). The total current population in Sweden is 10 million. At present, the information in SKaPa covers about 90% of the population of children and adolescents (0–19 years), and 47% of the population aged 20 years and older. To date, only 28 patients have opposed registration or have requested to have their dental information deleted from the registry.

**Table 1:** Number of affiliated DCOs and accumulated number of patients included in the SKaPa DW during the time period 2009–2018

Year	DCOs (*n*)	Patients 19 years and younger (*n*)	Patients 20 years and older (*n*)	Total (*n*)
2009	2	104,684	156,527	261,211
2010	3	148,779	241,753	390,532
2011	6	374,990	601,513	976,503
2012	12	1,173,812	1,826,414	3,000,226
2013	16	1,446,882	2,249,957	3,696,839
2014	20	2,147,271	3,161,581	5,308,852
2015	21	2,282,830	3,384,220	5,667,050
2016	21	2,456,200	3,640,947	6,097,147
2017	22	2,611,572	3,873,345	6,484,917
2018	22	2,768,484	4,164,843	6,933,327

DCO, dental care organisation.

## **Reports and access to registry data**

An annual SKaPa report^13^ is published. This contains data on caries and periodontal diseases, reported on aggregated national, regional, organisational and dental clinic levels. The report describes, for example, the patterns of dental visits, treatments and treatment outcomes, and oral health development.

The participating DCOs can directly access standard reports available online and compare their own clinical performance with results by others. Cross‐sectional and longitudinal data are provided that can be used for quality development of the care as well as for research purposes^14^. Upon request, customised de‐identified data outputs for single DCOs can be obtained, with detailed information on how data are retrieved.

Resources within SKaPa are allocated for education and tutoring in the use of registry data for quality development. To support such processes, SKaPa has developed a Manual for Quality Development in Dentistry in collaboration with the Qulturum Center for Learning and Innovation in Healthcare, Region Jönköping Council, Sweden (http://www.skapareg.se/vardutveckling). This manual is based on theories presented by Batalden and Davidoff^15^ and Deming^16^, and the Model for Improvement^17^ including the Ishikawa diagram^18^. Data access for scientific purposes is provided after a formal application to the SKaPa Scientific Review Board (http://www.skapareg.se/forskning). Approval by an ethics review board is mandatory. Several research projects, related to cariology, periodontics and pedodontics have obtained data from the registry (articles to be published).

## **Examples of data output related to the objectives for SKaPa**

The SKaPa concept is in line with contemporary ideas of systematic evaluation and continuous development of care^19^.

**Figure 2:**
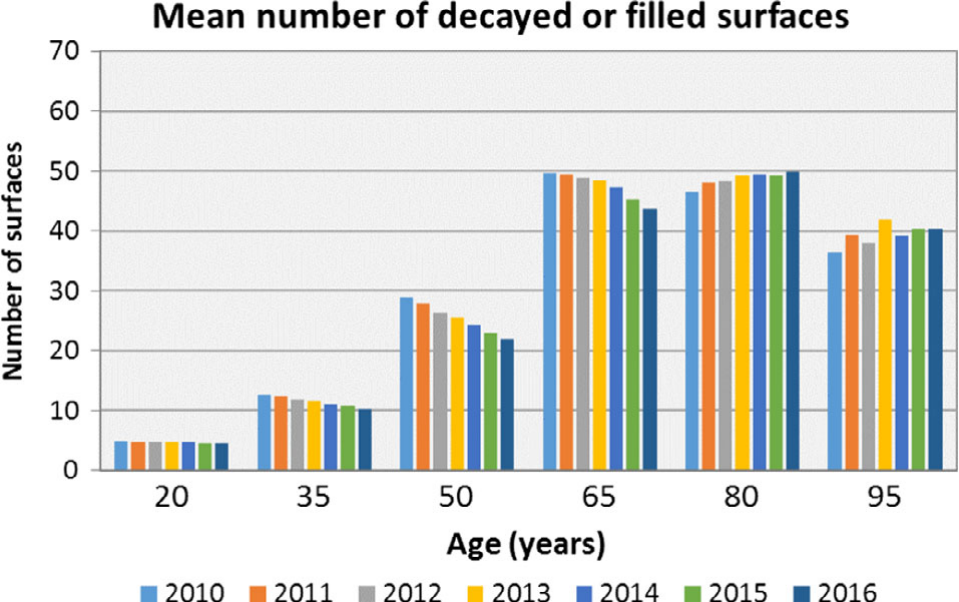
Mean number of decayed or filled tooth surfaces for individuals of different ages (third molars excluded). Time period: 2010–2016. Data are based on all individuals in the different ages with a comprehensive examination.

The SkaPa generates epidemiological data (objective 1). As an example, repeated cross‐sectional data on a national level of the prevalence of dental caries or fillings among individuals of different ages are presented for the years 2010–2016 in *Figure* 2. The data illuminate different patterns over the reported 6‐year period depending on age. While the mean number of tooth surfaces with caries or fillings remained stable in 20‐year‐old individuals, a decrease is evident in individuals aged 35, 50 and 65 years, and an increase in individuals aged 80 and 95 years.

**Figure 3:**
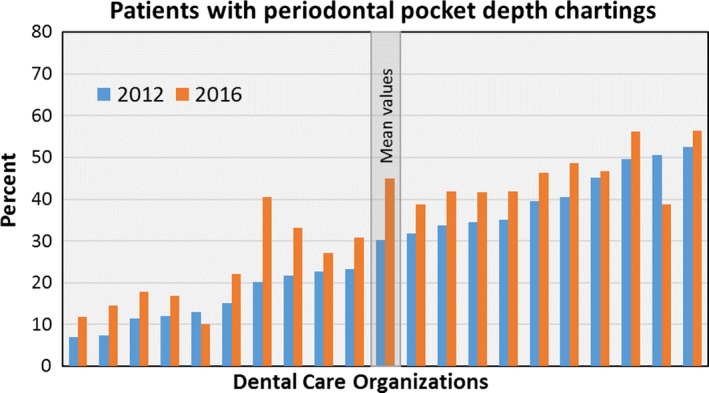
Percentage of patients 20 years and older with documented periodontal pocket depth of all persons with comprehensive oral health examination in the various dental care organisations (DCOs). Number of patients: 2012 *n *= 1,288,462 and 2016 *n *= 1,307,573.

While professional knowledge is essential, an understanding of quality improvement seems to be less well established within the profession, although it is important for a sustainable change to improve oral health^20^. SKaPa can deliver data as a base for improvement of quality in dental care (objective 2). An example is presented in *Figure* 3, which describes, for 2013 and 2016, the proportion of adult patients who received a comprehensive oral health examination including periodontal pocket depth registration. A general trend of an increased percentage in 2016 is evident, indicating an improved quality of care with regard to periodontal diagnosis. However, marked and unjustified differences between the DCOs remain, which call for skills development and compliance with guidelines in order to improve clinical routines and quality of care. SKaPa provides customised information for specific issues and expertise to support organisations or individual dental clinics in carrying out quality development projects^21^.

**Figure 4:**
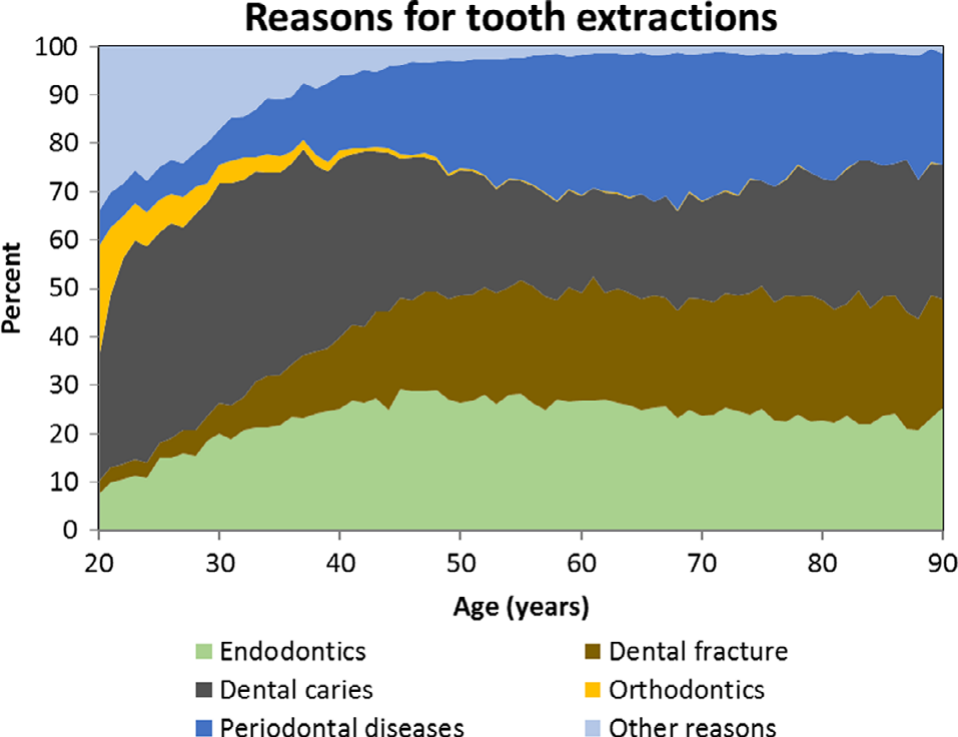
Percentage distribution of reasons for tooth extraction in 2016 in patients 20–90 years old grouped into six categories: Endodontic, Dental caries, Periodontal diseases, Dental fracture, Orthodontics, and Other reasons. Number of patients with comprehensive oral health examination in 2016, *n *= 1,337,145. Number of patients with one or more teeth extracted, *n *= 91,468. Total number of tooth extractions, *n *= 207,339.

Treatment decisions in dentistry are illustrated in *Figure* 4, which represents data on causes for tooth extractions among patients aged 20–90 years in 2016 (objective 3). While caries was the dominating cause in the young age groups, periodontal diseases were a more common cause for tooth extractions for persons 50 years of age and older. Endodontic reasons and tooth fractures together accounted for about 45% of all tooth extractions in individuals aged 45 years and above, an observation that in relation to potential for improved quality of care deserves a closer analysis. In the interpretation of these data, one has to be aware that a tooth may show more than one diagnosis/condition, but that the recording systems only allow for one to be recorded as a basis for the decision to extract a tooth.

**Figure 5:**
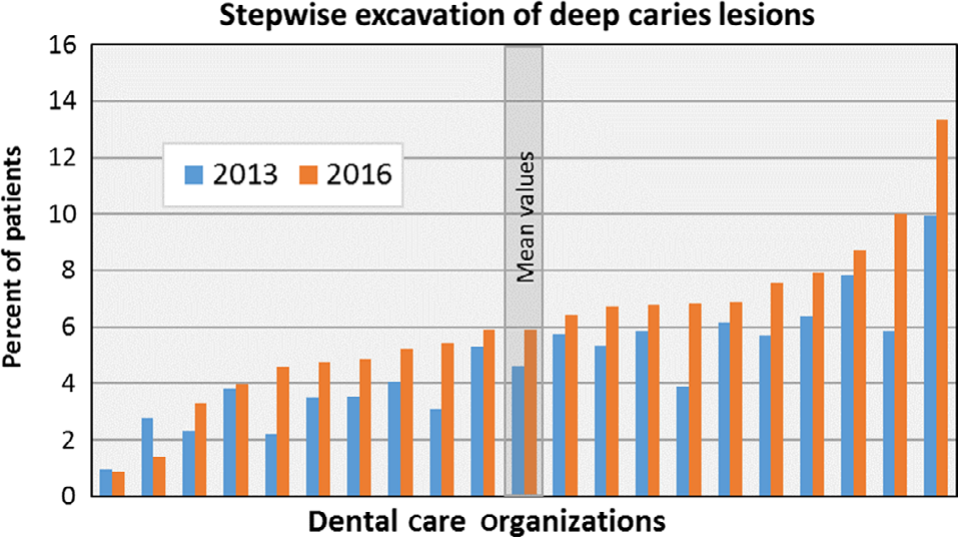
Percentage of patients 20 years and older who received stepwise excavation of deep caries lesions, of those with restorative therapy due to caries in 2013 (17,748/384,362) and 2016 (21,171/359,045), respectively, per dental care organisation (DCO).

For the evaluation of the implementation of treatment recommendations by national or regional guidelines^22^, ^23^, data generated by the SKaPa registry can be used (objective 4). This may be exemplified by data regarding stepwise excavation of deep caries lesions, which is an approach recommended by the Swedish national guidelines. Analyses of SKaPa data reveal an increased use of the method between 2013 and 2016 (*Figure* 5), but also marked differences between DCOs. A randomised controlled study with a follow‐up period of 1 year showed that stepwise excavation led to significantly fewer pulpal exposures compared with direct complete caries excavation^24^. It would be essential to analyse the external validity of the evidence from the randomised controlled study through long‐term follow‐up data. SKaPa enables such an evaluation by providing information on clinical consequences as well as health‐economic effects of the recommendation (objective 5).

**Figure 6:**
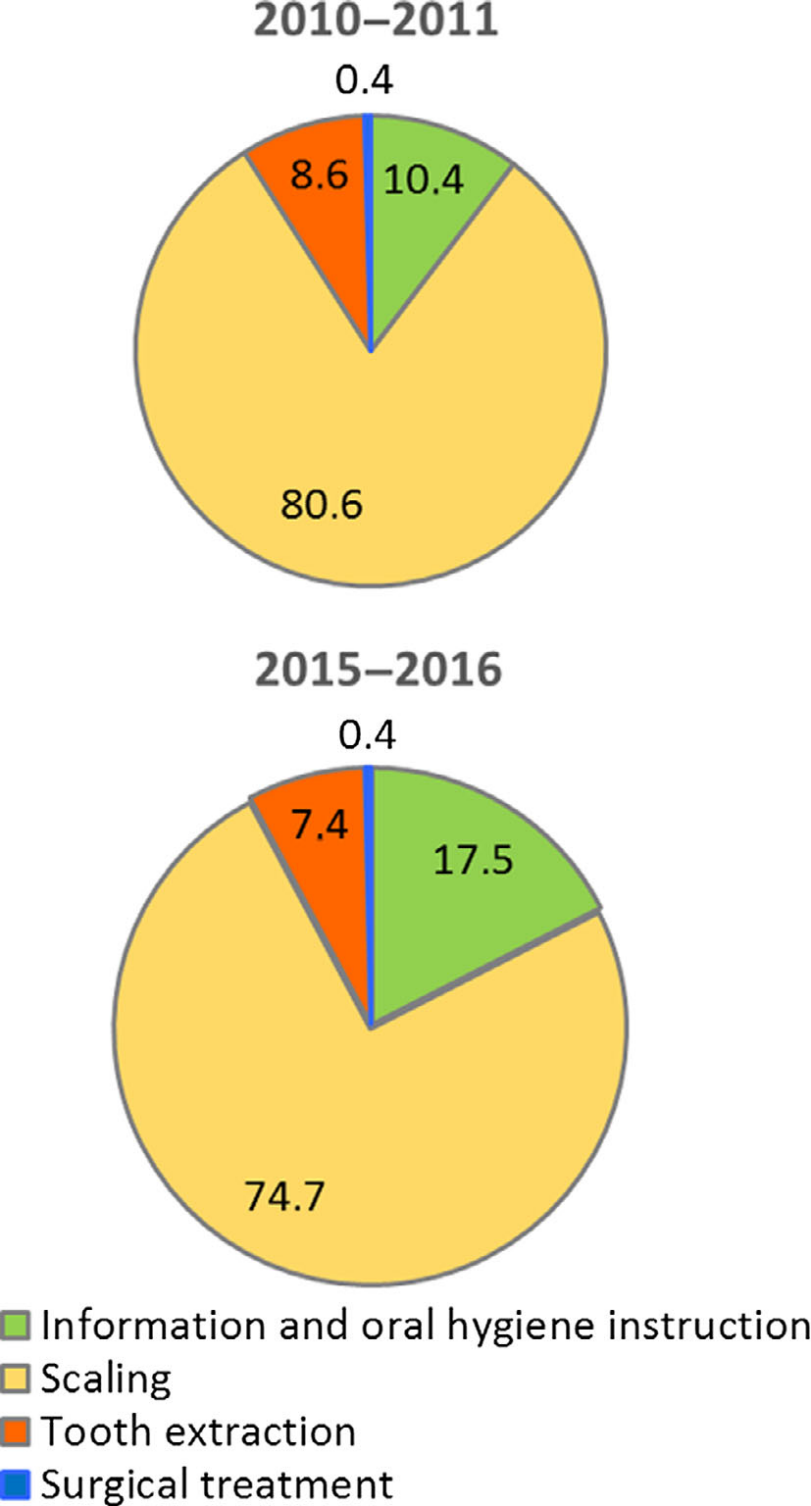
Percentage distribution of four main groups of treatment procedures related to periodontitis in patients aged 20 years and older: information and oral hygiene instruction, scaling and root planing, tooth extraction and surgical treatment. Total number of treatment procedures and treated patients during 2010–2011: 820,059/385,765; and 2015–2016: 1,102,479/477,012.

Another example related to the implementation of the Swedish national guidelines is the evaluation of the proportions of various treatment procedures provided to patients with periodontitis during two time intervals (2010–2011 and 2015–2016; *Figure* 6). The increase between the two time periods in the proportion of ‘information and oral hygiene instructions’ coincided with recommendations given in the national guidelines introduced in 2011 and an expansion in reimbursement by the dental care benefits system (TLV^7^).

Patient‐reported outcome measures (PROMs) have become important in the evaluation of healthcare, and all Swedish NQRs are obliged to present such data (objective 6). Currently, however, in most patient record systems in Sweden, PROM is limited to a single global question on patient‐perceived oral health, which has to be registered. There is thus an obvious need to develop effective and adequate methods to obtain more extensive PROM information that can be delivered to the registry. To meet this demand, SKaPa has initiated projects for further development of PROM in dentistry based on modern concepts of item response theory^25^ and the experience of PROMIS^®^ 26.

## **Discussion**

National quality registries are considered powerful tools in support of quality development of healthcare, promoting patient in favour of patient safety and health, and as sources for valuable and valid information for clinical research^3^. To the best of our knowledge, SKaPa is the first NQR in dentistry solely based on automatic retrieval and delivery of data from patient records. Developing the SKaPa concept in close dialogue with broad representation from the dental field, and basing the registry on automatic retrieval of data from patient records, were judged to be decisive factors for a successful development and implementation of the registry.

The SKaPa provides information from dental praxis with high external validity^27^. Accordingly, the registry offers a significant source of data for support of quality development of dental care, dental research and health‐economic analyses, as well as substantiation of scientific data from randomised controlled studies. SKaPa information may also reveal gaps in knowledge and identify issues to be further evaluated in intervention studies. The use of unique personal identifiers enables longitudinal studies and facilitates large‐scale registry studies interlinking oral health data with data from other registries, for example socio‐economic and general health registries. Such studies may provide important information about subgroups in the population^28^.

Data on dental care for all patients attending the affiliated DCOs will automatically be accessible for delivery to SKaPa. In 2018, the SKaPa database included information on about 90% of the Swedish population of children and adolescents (0–19 years), and about 50% of those aged 20 years and older. The lower percentage of adults is related to the fact that comparatively few private DCOs so far have been connected to SKaPa. This is a limiting factor with regards to information about middle‐aged and older adults in particular^29^. However, there is a great interest among private DCOs in being affiliated. The recently performed successful testing of delivery of data to SKaPa from one additional electronic patient record system, which is used by a large sector of private dental clinics, will lead to a marked increase in the number of affiliated private DCOs and the proportion of the adult population covered by SKaPa. Thus, SKaPa has the potential to become a registry that covers the vast majority of dental care related to caries and periodontal diseases in Sweden. There are no other registries, neither in Sweden nor in other countries to our knowledge, with similar potential and the capability to fulfil the SKaPa objectives. For that reason and for the purpose of making the SKaPa registry and its concepts known and available to the scientific community for potential research studies, this overview description of the registry is presented.

An important consideration in relation to the population coverage is that about 25% of the adult population in Sweden does not seek dental care on a regular basis^8^. Nevertheless, because SKaPa monitors both regular and non‐regular attendance, longitudinal and cross‐sectional epidemiological data produced by the registry may still be valid for a description of oral health conditions among the population in Sweden and potential differences in relation to, for example, demographics, geography or gender. SKaPa will thus serve as an important tool for targeting the WHO's vision to ‘promote oral health and reduce inequalities’^30^.

The fact that non‐calibrated examiners generate the diagnostic data may be of minor importance for the validity of data, because with the very high number of clinicians involved (currently almost 13,000), the probability of systematic errors should be low. A scientific study is currently being conducted to evaluate the effect of non‐calibrated examiners on the caries data registered in patients’ records and delivered to SKaPa. Because diagnosis and treatment procedures used in adult dentistry are clearly defined and regulated by the national reimbursement system^7^, high data reliability is likely. However, a limitation is that some treatment codes do not always discriminate between treatments on a detailed level, for instance various types of fluoride applications. Further, treatment data for children and adolescents might be less well documented in patient records as reimbursement is by capitation. However, there is a clear trend towards the use of the TLV codes for documentation of treatments also in children and adolescents, which will improve the quality of output data.

The design of the dental record systems is essential to the documentation of dental care and data quality. Initially, data quality controls revealed some shortcomings in the patient record systems that required correction, but subsequent regular quality controls have not revealed any decisive weaknesses. The regular quality controls, as well as continuous development of new parameters, necessitate a dynamic process with close collaboration with the providers of the dental record systems to secure a high level of information quality.

The SKaPa offers exciting future possibilities. The large datasets in SKaPa (‘big data’) make it possible to use data mining^31^. Currently, this has only been used to a limited extent in dental research^32^. The SKaPa data warehouse provides the opportunity to explore patterns of oral diseases and dental care previously unidentified, and to reveal noteworthy associations^33^. Because of its nationwide coverage, trends and changes in treatment strategies can be identified, visualised and analysed^34^. Another future prospect is to make it possible for the caregiver to present such information chair‐side to the patient as a background for treatment proposals^35^. Such information may even include variables for each individual patient.

## **Conclusion**

The SKaPa concept adheres to a new era of evaluation of everyday dental healthcare and conducting clinical and epidemiological research. As a national registry based on automatic retrieval of data directly from patient records, it creates access to ‘big data’ on a national level. Description of oral health, evaluation of national guidelines, clinical programs and patient‐reported outcomes, as well as external validation of clinical evidence generated by randomised controlled trials and health‐economic evaluations, can be performed as a base for new or adjusted policies in dentistry. The SKaPa concept thus offers a unique tool as support for the improvement of oral health and quality of dental care.

## **Conflict of interest**

The authors received no financial support, and declare no potential conflicts of interest with respect to the authorship and/or publication of this article. The authors alone are responsible for the content and writing of this paper.
